# Treatment of rheumatic diseases and hepatitis B virus coinfection

**DOI:** 10.1007/s00296-014-3195-8

**Published:** 2014-12-31

**Authors:** Anna Felis-Giemza, Marzena Olesińska, Katarzyna Świerkocka, Ewa Więsik-Szewczyk, Ewa Haładyj

**Affiliations:** Institute of Rheumatology, Connective Tissue Department, 1 Spartanska Street, Warsaw, Poland

**Keywords:** Rituximab, Tocilizumab, Abatacept, Biological therapy, Hepatitis B, Rheumatic diseases

## Abstract

We often encounter rheumatological patients coinfected with hepatitis B in daily practice. In this paper, we will discuss the basic characteristics of the virus of hepatitis B, course of infection, the safety of rituximab, tocilizumab, abatacept treatment and therapeutic recommendations in management of patients with rheumatic diseases.

## Introduction

The introduction of biological agents significantly increased the efficiency of the treatment of rheumatic diseases and limited permanent damage to organs in the course of the inflammatory process. Particular efficacy is achieved by depletion of B cells the monoclonal anti-CD20 (rituximab), antagonism of interleukin-6 (IL-6R) (tocilizumab) and inhibition of T cell activation molecule, CTLA-4-Ig (abatacept). These methods allow for the effective treatment of a growing number of patients with active forms of rheumatoid arthritis (RA), juvenile idiopathic arthritis (JIA), psoriatic arthritis (PsA) and ankylosing spondylitis (AS).

Coexistence of severe rheumatoid disease with chronic viral hepatitis (hepatitis) type B makes the treatment of abatacept, tocilizumab or rituximab risky because it can lead to reactivation of viral infection.

Knowledge of areas endemic for hepatitis infections, increased risk groups, the natural history of infection and the impact of the treatment of rheumatic diseases on the course of hepatitis increases the effectiveness and safety of treatment in patients eligible for the treatment of biological agents.

## Hepatitis B

### Hepatitis B virus (HBV)

HBV belongs to the family Hepadnaviridae and comprises a circular double-stranded DNA and DNA polymerase surrounded by the core antigen (HBcAg) [1]. The HBcAg consists e antigen (HBeAg). HBcAg is surrounded by a shell containing the lipoprotein surface antigen (HBsAg) [1, 2].

Important features of HBV infection affecting the course of HBV DNA are the ability to integrate into the DNA of infected hepatocytes and the replicative capacity. It is estimated that the chronically infected blood stream is expelled section approximately 1011 viral particles. Of particular note is the fact that the HBV reverse transcriptase has a low error correction capability of reading, resulting in a high rate of mutation [3].

Areas endemic for HBV infection are a southeast Asia, Africa and other regions of the world outside North America and Western Europe, and Australia [7]. In Poland, 1.5 % of the population is chronically infected with HBV, which is about 700 thousand [8].

### Course of HBV infection

HBV infection can occur in a variety of clinical forms—from asymptomatic, by explicitly acute viral hepatitis, chronic inflammation, which can result in cirrhosis and liver cancer [1, 2, 4]. The majority of adult patients (>95 %) of host defense mechanisms allow for total eradication HBV [12].

There are the following phases of HBV infection [5, 6]:

1. Immune tolerance phase—due to the low immune response, aminotransferase levels are low, and inflammatory changes in liver histopathology not significantly increased despite a high viral load of HBV DNA (106–109 copies/ml). If this occurs during the integration of HBV DNA with the host DNA in hepatocytes, the likelihood of HBV eradication decreases—both spontaneous and induced by treatment [12];
2. Immune clearance phase—active immune response against HBV-infected hepatocytes, leading to an elevated aminotransferase levels and severe necro-inflammatory changes in liver histopathology. HBs antigen is always present, and HBe antigen—positive or negative—antibodies to HBe may be present. HBV DNA viral load is moderately high (105–107 copies/ml). The majority of patients in this phase (about 90 %) present a spontaneous loss of HBeAg antigen and the appearance of antibodies to HBeAg, called seroconversion [12];
3. Immune control phase—after HBeAg seroconversion antibodies to HBe are present, HBV DNA replication is low (<105 copies/ml), transaminase activity is normal or slightly increased, and the histopathological changes in the liver are absent or minimal;
4. Phase reactivation of infection—dominate the mutant HBV DNA, which do or emit a small amount of HBe antigen. The observed increase in serum HBV DNA viral load, increased transaminases and liver biopsies are present necro-inflammatory changes.

In the initial period of hepatitis B infection, which lasts several weeks, HBV remains hidden from the immune system of the host. Then, a strong innate (NK cytotoxic lymphocytes and NKT—natural killer—natural killer T) and acquired (mainly CD8 + T cells) immune response is directed against infected hepatocytes secreting antigens HBV [9]. The virus removal process involves many cytokines, such as interferon-α (IFN-α), interferon-β (IFN-β), interferon-γ (IFN-γ), tumor necrosis factor-α (TNF-α) or interleukin-6 (IL-6) [9–11].

### Treatment of hepatitis B

Currently, the treatment of chronic hepatitis B, the classical recombinant IFN-α and its pegylated form, and nucleoside and nucleotide analogs, such as lamivudine, adefovir, tenofovir and entecavir, result in the suppression of HBV replication. In case of the selection of strains resistant to lamivudine, adefovir, tenofovir or entecavir may be used [8].

### HBsAg carrier state

After HBeAg seroconversion, patients can go in HBV carrier state. At this stage, the disease is characterized by the presence of HBsAg in the serum, low or undetectable levels of HBV DNA, normal aminotransferase activity and lack HBeAg [13]. Fewer than 1 % of inactive HBV carriers per year will undergo spontaneous seroconversion HBsAg [14]. In the carriers of HBsAg, the use of immunosuppressive therapies can lead to reactivation of infection.

### Hidden HBV infection

Hidden HBV infection can be described as the presence of HBV DNA in patients without HBsAg, and with or without anti-HBc and/or anti-HBs. In patients with the presence HBV and HBsAg is undetected, HBV DNA viral load is low, even of the order of several virus copies per milliliter of plasma or liver tissue, impossible to detect with a serological test and detectable by molecular tests. Another reason for latent HBV infection may be a mutant HBV infection, which is not recognized by the serological test.

### HBV reactivation

 This reactivation of HBV hepatitis occurs during immunosuppressive therapy or shortly after its cessation, with concomitant increase in the levels of HBV DNA (≥tenfold increase) or the absolute height of more than 9 log 10 copies/ml or sudden twofold increase in the activity of alanine transaminase (ALT) baseline and fivefold with respect to the upper limit of normal value or greater than 300 IU/L. It needs exclusion of delta virus superinfection, acute infection with HAV, HCV, HEV, toxic hepatitis and other liver diseases of other than viral atiology [15, 16]. To monitor HBV reactivation, the level of HBV DNA and ALT activity should be monitored. The increase in HBV viral load always precedes an average of 2–3 weeks growth of ALT activity [17]. Reactivation of infection may occur without any clinical signs (only increase of HBV DNA, the presence of HBsAg), reveals as increase in liver transaminases, or even jaundice until the fatal fulminant hepatitis patients [18].

Immunosuppressive agents by inhibiting host immune responses lead to enhanced replication of HBV in the liver and to enhanced expression of the viral antigenic epitope [19]. Hepatitis may occur both during immunosuppressive therapy and thereafter, because of the renewal of the immune response against viral infected hepatocytes [20]. Also the use of glucocorticoids is associated with an increased risk of HBV reactivation [17].

Glucocorticoids (GKS) are well-known risk factor for infection by inducing immunosuppression. The degree of suppression of the immune system increases with dose and duration of treatment. For the treatment of longer than 2 weeks, the dose above 20 mg/day of prednisolone or its equivalent is generally considered clinically significant to induce immunosuppression [37], but has been shown that the cumulative dose of 500 mg or below the average daily dose of less than 10 mg of prednisolone does not increase the risk of infectious complications, and can be stated that are potent immunosuppressive [38]. GKS are usually served with disease-modifying drugs (DMARDs) or TNF-α inhibitors in the treatment of rheumatic diseases, so that data on monotherapy are limited [39]. GKS influence on HBV reactivation cannot be denied. In 30 of 144 patients (one with a history of infection and the other with chronic HBV), HBV reactivation occurred after a median time of 9.8 months. HBV markers in serum were determined before steroid therapy in 67 patients, of whom one was a history of HBV infection and the remaining patients had chronic infection. In addition, Bae et al. [39, 40] reported a case of fatal hepatitis B, and it was a reactivation of infection during long-term, low-dose treatment with GKS of inactive carrier of HBV. Most of the patients treated in combination with glucocorticosteroids DMARDs (sulfasalazyna and chloroquine), but HBV reactivation was mainly associated with glucocorticoid therapy. There are two described cases [38, 39] of patients with RA-treated GKS alone. None of the patients was described prophylactic antiviral therapy. In both cases, HBV reactivation occurred in 5 and 9 months.

### Non-biological disease-modifying drugs (nb-DMARD)

Based on previous data, it can be concluded that the non-biological DMARDs treatment of patients with serological profile HBsAg (+)/HBsAg (−) and anti-HBc (+) may increase the risk of reactivation of HBV. Non-biological DMARD therapy is relatively safe in patients with a low risk of reactivation—a low level of HBV DNA, untreated GKS, anti-HBs (+) and the use of prophylaxis in these patients are not recommended. In the group of 224 patients treated with nb-DMARDs, 192 underwent HBV infection and 32 had diagnosed with chronic hepatitis B [38]. Only four patients with chronic hepatitis B received prophylactic antiviral therapy. HBV reactivation was observed in 10 patients (4.46 %) with a median follow-up of 13.2 months [42–46]. Methotrexate (MTX) was administered to eight (out of ten) in patients with HBV reactivation [42–46]. Antiviral therapy was started after HBV reactivation. Efavirenz was the preferred drug in most of the patients. In one case, death of the patient with fulminant hepatitis after a 28-month follow-up was reported, despite treatment with IFN-β. There are also isolated reports of HBV reactivation during treatment MTX [41, 49–52], leflunomide [44], azathioprine [52], chloroquine [53] and sulfasalazine [44]. At the same time, there are early reports on the role of sulfasalazine in the intensification of apoptosis of cells secreting antigen HBV [48]. There is no evidence of HBV reactivation in patients treated with gold agents and d-penicillamine.

## Biological DMARDs

### Anti-TNF α agents

TNF-α molecule is a homotrimer, consisting of three units of a weight of 17 kDa each [57]. TNF-α in RA patients is synthesized in the joints synovium, blood vessels and articulate pannus [58]. High levels of TNF-α in joint space stimulate osteoclasts, which lead to bone resorption [58]. The use of biologics in RA patients coinfected with HBV or HCV is not fully secure. Although TNF-α inhibitors did not show hepatotoxicity, their use carries the risk of activation or exacerbation of hepatitis [59].

TNF-α inhibits the replication of HBV DNA. Low concentrations of TNF-α abolish its desired biological effect. The lack of balance between the concentration of TNF-α and interferon-γ may reduce the effectiveness of the elimination of the HBV [60–62].

It is believed that inhibition of TNF-α decreases the inflammatory cell response against a viral infection, and viral replication is extended and prevents the destruction of infected cell [63].

The risk of reactivation of HBV during therapy with anti-TNF-α increases. Therapy with anti-TNF-α may cause HBV reactivation in patients with HBsAg (+) or HBsAg (−) with detectable HBV DNA and additionally with the status of HBcAg (+) and HBsAg (−). At the same time, treatment of anti-TNF-α can be used safely in patients with HBsAg (+) with undetectable DNA HBV [64]. The current long-term observation of patients with a serological profile HBsAg (−) and anti-HBc (+) treated with anti-TNF-α suggests that the percentage of reactivation is low. This confirms the cohort study 72 patients observed from 43 to 21 weeks, in which no reactivation was observed [65].

In a study by Perez-Alvarez et al. [66], 257 patients were analyzed—89 carriers of HBsAg and 168 hidden carriers (anti-HBc+) treated with anti-TNF-α due to rheumatoid arthritis or inflammatory bowel diseases. Among carriers of HBsAg, HBV reactivation was observed in 35 (39 %), including acute liver failure in 5 (5.6 %). The incidence of HBV reactivation was higher in patients under immunosuppressive treatment (96 vs. 70 %, *p* = 0.033) and lower in patients who received antiviral prophylaxis (23 vs. 62 %, *p* = 0.003). In the hidden HBV carriers, reactivation was observed in 9 (5 %) patients, including one patient developed acute liver failure.

Data in the literature concerning the reactivation of infection during treatment with anti-TNF-α show that infliximab often causes HBV reactivation as compared with etanercept. More often, the reactivation of these infections occurs during the third dose administration [67, 68]. Greater probability of HBV reactivation during treatment of infliximab than that of etanercept can be explained by pharmacological and biochemical differences between these molecules. HBV reactivation associated with infliximab is more frequent in HBV carriers compared with those infected with concealed HBV [66]. Etanercept has a shorter half-life than infliximab, which allows for faster elimination of the drug after cessation of treatment. In case of infliximab discontinuation, the rebirth of an increased pool of macrophages and T lymphocytes occurs, resulting in an acute response in relation to replicating virus, which can potentially cause damage to the fulminant hepatitis [69]. Thimm et al. [70] studying hepatitis B in animals have found that several months after the depletion of CD8 + T cells, there was an unexpected increase in the number of these cells to baseline. He was accompanied by an increase in liver enzymes and increased the elimination of HBV DNA without the formation of neutralizing antibodies anti-HBs. These studies suggest that elimination of the HBV DNA is associated with restoration previously eradicated CD8 + cells [70]. The combination of infliximab with methotrexate therapy can reduce the elimination of CD8 + T lymphocytes specific for HBV present in the hepatocytes [20]. HBV reactivation may appear during infliximab monotherapy [71]. As infliximab concentration decreases, the risk of exacerbation of HBV infection increases, because the remaining CD8 + T cells respond to the viral replication, previously inhibited by the action of TNF-α [72].

### Rituximab

Rituximab is a chimeric human–murine monoclonal anti-CD20 antibody used in the treatment of rheumatoid arthritis and other rheumatic diseases such as vasculitis. Data on the safety of rituximab in patients with rheumatic diseases and HBV are scarce, but study on patients treated for hematological malignancies indicates a high risk of HBV reactivation in patients undergoing no prophylaxis (27–80 %) [21–23]. Four cases of HBV reactivation were described in patients with RA treated with rituximab [24, 33–35]. In two patients with chronic hepatitis B treated with rituximab with prior prophylaxis, no HBV reactivation was described [25]. The risk of HBV reactivation increases with concomitant use of corticosteroid or chemotherapy [26]. To date observations of patients with hematologic malignancies showed that the HBV reactivation can occur with rituximab alone [26, 27]. There are also reports that show the opposite—rituximab alone HBV reactivation risk is greater in comparison with the same chemotherapy [26, 28].

### Abatacept

Abatacept is a fusion protein obtained by genetic engineering. It consists of the extracellular domain of human CTLA4 and a modified Fc fragment of human immunoglobulin G1 for preventing the complement fixation. Abatacept, in contrast to TNF-α inhibitors, directly neutralizes pro-inflammatory cytokines, but specifically inhibits T cell activation in recent retrospective study described in eight patients with chronic hepatitis B treated with abatacept, respectively, four of them received prior anti-viral prophylaxis. In all patients who did not receive antiviral prophylaxis, HBV reactivation occurred [32]. One case of a patient treated with abatacept was described, with a serological profile HBsAg (−), HBeAg (−), anti-HBc (+), anti-HBs (−) and anti-HBe (+) during qualifying for the therapy. After 10 months of treatment, HBsAg (+) and HBV DNA were detected, hepatitis B was diagnosed, abatacept was stopped, and successful treatment with lamivudine was entered [28]. In second case, serological profile was HBsAg (−), HBeAg (−), anti-HBc (+), and anti-HBs (+); serum HBV DNA became undetectable 4 months after initiation of tenofovir treatment [34].

### Tocilizumab

Tocilizumab (TCZ) is a humanized monoclonal antibody directed against the receptor for interleukin 6. RA patients coinfected with HBV were not included to clinical studies with tocilizumab. For this reason, data on the safety of tocilizumab therapy in patients infected with HBV are scarce. Signaling pathway inhibition effect of IL-6 on the course of HBV infection has not been investigated; however, IL-6 is associated with both liver damage in the course of HBV infection and virus removal [37]. Two cases of patients who received tocilizumab with hepatitis B treated with antiviral prophylaxis were published in Japan, and no HBV reactivation was reported [29–31]. Therefore, in patients with chronic hepatitis B and normal liver function, antiviral therapy should be considered before treatment with tocilizumab. Prevention is also indicated in HBsAg carriers and in patients with hidden hepatitis B. Nakamura [47] study of 18 patients treated TCZ with a serological profile HBsAg (−)/HBsAg (+) anti-HBc (+), in two patients HBV reactivation was observed.

### Antiviral prophylaxis

Currently, there are no formal guidelines on eligibility for treatment of HBV patients with rituximab, abatacept and tocilizumab [35]. In these patients, it is recommended the initial determination of the presence of HBsAg and anti-HBc and anti-HBs. Patients with current Hbs antigen should receive antiviral treatment. Available data on this subject for rituximab in rheumatic diseases are limited, and for tocilizumab and abatacept inaccessible.

In each patient eligible for the treatment of anti-TNF-α, HBsAg and anti-HBc should be determined [73–75]. Patients with present isolated anti-HBc must also make a determination of HBV DNA by PCR. The carriers of HBsAg should monitor the activity of transaminases and viremia up to 3 months after completion of therapy medicines called anti-TNF [75].

Antiviral therapy for HBV infections led to a reduction in viral load, in spite of chronic drug therapy from the group of anti-TNF-α. Antiviral therapy may be implemented either preventively, at the same time as the anti-TNF-α or to be reserved for cases in which there is an viremia increase [76]. It should be borne in mind that the development of HBV mutations during the course of therapy leads to resistance to antiviral therapy and the development of severe liver inflammation [69]. It is recommended that patients with potential risk of belonging to the group of “hidden carriers,” before treatment with anti-TNF-α, should carry out detailed analysis of the clinical and laboratory signs of HBV reactivation (including the determination of HBs antigen) [77].

The HBsAg carriers and hidden HBV carriers before inclusion of anti-TNF-α should use prophylactic antiviral treatment. This is confirmed by a randomized study of patients undergoing chemotherapy [69, 78, 79], while in patients with rheumatic diseases, several clinical cases and reviews were performed [66, 80–85].

Based on the review of the published cases of patients with rheumatic diseases treated with anti-TNF-α (*n* = 255), made by Ramos-Casals in 2010, it can be concluded that the risk of HBV reactivation is approximately 38 % (*n* = 87) in patients HBsAg (+), without preventive antiviral treatment [80]. There are lack of reliable data on the safety and efficacy of antiviral treatment as protection against HBV reactivation [86]. Zingarelli et al.’s [87] literature review estimated that antiviral prophylaxis with lamivudine in HBsAg (+) patients reduces the risk of reactivation (lamivudine 14 % vs. without prophylaxis 69 %). Prophylaxis may be considered in the later treatment with antiretroviral agents with higher genetic barrier, with a low risk of viral resistance and a higher efficacy (tenofovir or entecavir). During therapy, patients should be monitored for HBV DNA (every 3–6 months) and ALT (every 3 months) [19]. These drugs should be discontinued after 6–12 months after treatment of anti-TNF-α [20, 21]. In conclusion, tenofovir or entecavir is a good choice among active carriers with high HBV DNA in the case of reactivation or development of hepatitis, while lamivudine is recommended as prophylaxis in inactive and hidden carriers [21, 22].

The 2008 guidelines of the American College of Rheumatology (ACR), updated in 2012, do not recommend the use of anti-TNF-α in pharmacologically untreated chronic hepatitis B and treated with considerable damage to the liver (Child class-Pugh B and C). There are no clear recommendations on their use in adjusted cirrhosis (Child–Pugh class A). The novelty in relation to the recommendations of the ACR 2008 is to allow the possibility of the use of etanercept in the treatment of RA patients with hepatitis C [74].

Anti-TNF-α should not be used in patients with chronic HBV infection, according to the guidelines European League Against Rheumatism (EULAR). If during treatment with anti-TNF-α patient is diagnosed with hepatitis B, you can implement an antiviral drug.

The prevention can be considered for treatment with newer antiretroviral agents with higher genetic barrier with a low risk of viral resistance and higher efficiency (tenofovir or entecavir). During therapy, patient should be monitored for the presence of viral DNA (every 3–6 months) and ALT (every 3 months) [28]. These drugs should be discontinued after 6–12 months after the completion of biological treatment [36–38].

Observational studies in oncology suggest extending the prophylaxis to the 12th month after the discontinuation of immunosuppressive treatment [55]. In case reports of patients treated with rituximab, HBV reactivation occurred later [53, 54]. According to the onco-hematological recommendations, prior to starting RTX treatment, all patients should be screened for HBV infection. While HBsAg-positive active carriers should receive long-term antiviral treatment with entecavir or tenofovir, inactive carriers are candidates for universal prophylaxis with lamivudine, or entecavir or tenofovir in selected cases, to prevent hepatitis reactivation. Conversely, for HBsAg-negative anti-HBc-positive carriers, that is, those with resolved HBV infection, universal prophylaxis with lamivudine is recommended for those with onco-hematological diseases, whereas watchful monitoring of HBsAg/HBV DNA levels is advisable for all the other indications [56].

In conclusion, tenofovir or entecavir is a good choice among active carriers with high HBV DNA, in the case of reactivation or development of hepatitis, while lamivudine is recommended as prophylaxis in inactive and hidden carriers [38, 39]. It should be emphasized that the final diagnosis of hepatitis and implementation of antiviral treatment should be carried out in close collaboration with a specialist in the field of infectious diseases.

## Summary

Available data based on a small number of case reports in the literature are not always consistent. Therefore, before starting treatment HBsAg, anti-Hbc, and anti-Hbs should be determined, especially in people at increased risk. In patients with positive tests for the presence of virus, it is recommended to consult with physicians experienced in the treatment of viral hepatitis. Throughout the period of biological treatment and a few months after treatment, patients should be closely monitored for signs and symptoms of active HBV infection and, if necessary, take appropriate treatment. The risk of reactivation of hepatitis B appears to be large, and it can reduce the prophylactic use of lamivudine.
